# Nursing Students’ Use of Digital Resources for Self-Directed Learning in Bioscience

**DOI:** 10.1177/23779608251363870

**Published:** 2025-07-29

**Authors:** Victoria Oppegaard Berre, Unni Knutstad, Kari Toverud Jensen

**Affiliations:** 1 Faculty of Health Sciences, 60499 Oslo Metropolitan University, Oslo, Norway

**Keywords:** Nurse, education, multimedia, anatomy, physiology, digital, learning

## Abstract

**Introduction:**

First year nursing students often find bioscience challenging to learn and understand, leading many students to seek additional support through digital resources. Previous research highlights students’ preference for flexible digital resources such as educational videos. However, researchers seem to raise critical reflections on the pedagogical value of digital resources, particularly how they can support, enhance or improve learning. Self-directed learning is essential in higher education and particularly important in health professional education as it entails lifelong learning, crucial to ensure safe and efficient patient care. Employing the cognitive theory of multimedia learning, this study offers insights into how digital resources may support self-directed learning in bioscience among first year nursing students.

**Objective:**

To explore how digital resources can support self-directed learning among first year nursing students in bioscience. In this study, the term “digital resources” encompasses students’ use of online platforms that offer educational videos, interactive exercises and personalized feedback, and their use of internet searches. Framed within the concept of self-directed learning, the researchers explore how students engage with such digital resources outside of formal teaching settings.

**Method:**

A qualitative method with semi-structured individual interviews was employed. The data were analysed using Braun and Clarke's reflexive thematic analysis, with NVivo being used to organize the data into codes and themes. COREQ was applied as the reporting checklist.

**Results:**

Two main themes were identified: ‘Digital resources provide an overview over bioscience’, and ‘Motivational issues – deep learning or passing the exam’.

**Conclusion:**

Digital resources can support self-directed learning by guiding students in selecting content and providing a sense of control over the learning process.

## Introduction

Promoting lifelong learning among higher education students is a key priority in educational policy and practice, as outlined by the Bologna Process (Biggs & Tang, 2011). Lifelong learning has gained increased attention in connection with the digitalisation of higher education, which changes the ways in which lifelong learning is accessed (Lindqvist et al., 2024). For students to succeed with lifelong learning, it is of great importance to equip them with self-directed learning skills, as such skills may empower them to purposefully adapt to challenges throughout their educational and professional lives. Self-directed learning is particularly important for students in health professions, as they are expected to continuously update their knowledge and skills in clinical practice to ensure safe and appropriate patient care (Taylor et al., 2023; Wong et al., 2021). Knowles (1975) offers a frequently cited definition (Boyer et al., 2014; O'Shea, 2003; Saks & Leijen, 2014; Taylor et al., 2023) for self-directed learning, understood as ‘a process by which individuals take the initiative, with or without the assistance of others, in diagnosing their learning needs, formulating learning goals, identifying human and material resources for learning, choosing and implementing appropriate learning strategies, and evaluating learning outcomes’ (Knowles, 1975, p. 18). Given the focus of this study on students’ use of digital resources, the researchers recognize the importance of understanding self-directed learning in relation to the evolving digital landscape. Duval et al. (2017) contribute to an understanding of self-directed learning in the context of digital resources by emphasizing students’ responsibility in deciding what to learn, and where to find learning materials. In the literature, various terms such as self-directed learning and self-regulated learning are often used in parallel to describe the processes of student-initiated learning (Saks & Leijen, 2014). Noteworthy, Duval et al. (2017) use self-regulated learning. In this article however, the author team acknowledge that there are distinctions between these two terms as described by several researchers (Gandomkar & Sandars, 2018; Jossberger et al., 2010; Saks & Leijen, 2014; Taylor et al., 2023). While self-regulated learning, often referred to as the “micro-level”, involves regulatory skills at a task level, such as orienting one's learning toward a specific goal (Jossberger et al., 2010), typically contextualized within a classroom setting (Saks & Leijen, 2014). In contrast, self-directed learning is considered at the “macro-level”, building on the concept of self-regulated learning but extending beyond the task level. It involves broader planning, learner initiative and emphasizes learners’ responsibility, a desire to learn and conscious engagement with the learning process (Jossberger et al., 2010; Saks & Leijen, 2014). Moreover, self-directed learning is often used to describe learning activities that occur outside formal lectures. Therefore, the term self-directed learning is used in this paper as it reflects the focus on students’ self-initiated use of digital resources for learning bioscience outside the classroom settings, and how the literature portrays links between self-directed learning and lifelong learning. However, this paper also draws on literature related to self-regulated learning as there are a limited number of studies exclusively addressing self-directed learning with digital learning resources in bioscience. This is often the case in self-study research, as the two terms are often used synonymously in the literature (Saks & Leijen, 2014).

## Review of Literature

First year nursing students are expected to acquire extensive knowledge about bioscience: the human body's anatomy, physiology, and biochemistry, within the first four months of their university programme in Norway. Yet, nursing students often find bioscience challenging to understand (Jensen et al., 2018;; Barton et al., 2021; Bingen et al., 2019; Grønlien et al., 2021; McVicar et al., 2014; Montayre et al., 2021).

Within bioscience in nurse education, self-directed learning can be associated with metacognitive learning (Berre et al., 2024). Duval et al. (2017) emphasized that higher education students, especially younger students, often require support from the educational systems to develop the metacognitive skills needed to effectively engage in self-directed learning with digital resources on their own. In bioscience in nurse education the majority of students are young and lack prior experience with patient care, which may be a barrier for their learning process (Knutstad et al., 2021). According to Duval et al. (2017), technology has the potential to enhance and support what and how students learn, utilizing the power of interactivity and engagement in digital learning resources. Jensen et al. (2018) found that even though digital learning resources often emphasize student engagement and satisfaction, achievements in bioscience have not significantly improved. Similarly, Johnston et al. (2018) argue that although most of today's nursing students are ‘digital natives’, technology-based learning does not automatically mean effective and student-centred learning. The notion that technology does not automatically lead to improved learning itself is also supported by recent research, which emphasizes the importance of integrating digital technology within a holistic learning design grounded in pedagogical principles to enhance students’ professional development (Korseberg & Stalheim, 2024). Other researchers support this view by highlighting the importance for education to rethink course structure and the ways learning is supported in light of digitalisation in educational settings (Cramarenco et al., 2023). Zlatkin-Troitschanskaia et al. (2021) argue that as the digital landscape offers a wide range of possible learning platforms, it is important for students to develop skills to use appropriate resources. Harasim (2017) points out the opportunities technology holds for scaling education and deliver content to larger populations. However, qualitative aspects such as how learners engage with and make meaning from digital resources remain little explored (Harasim, 2017). Several scholars highlight critical reflections upon the use of digital technology in education, namely in what ways use of technology can support, enhance or improve learning (Duval et al., 2017; Grainger et al., 2023; Harasim, 2017). In this regard, qualitative research plays a valuable contribution to the field of self-directed learning with digital resources by exploring factors that students’ experience supportive in their learning processes.

In this study, the researchers understand digital resources as learning materials that are available through digital technologies. The digital resources addressed in this study are those that emerged from the data as particularly meaningful and relevant to the students’ learning process:

- Students’ use of online platforms that provide educational videos and interactive anatomical exercises, quizzes and personalized feedback
- Students’ use of internet searches, such as Google and medical encyclopaedias

Previous research on self-directed learning in a digital context among nursing students studying bioscience highlights findings on how resources may be supportive of self-directed learning. Egilsdottir et al. (2021) explored a set of digital learning resources together with students in a bioscience course in nurse education. The authors found that digital resources within nursing curriculum seem to create more self-directed learning spaces among students, allowing them to access and use the resources when they needed. Additionally, they found that digital resources seemed to help bridge the gap between theory and practice for nursing students. The authors highlight a need for further exploration of how digital learning resources can support students’ learning processes. Similar with the finding of Egilsdottir et al. (2021) regarding self-directed learning spaces, Montayre and Sparks (2018) found that nursing students preferred online video streaming over required textbooks. Moreover they found that the students preferred to visualize and listen to bioscience learning material, and appreciated the accessibility and flexibility with digital resources (Montayre & Sparks, 2018).

There are relatively few studies on self-directed learning in bioscience that have taken an exploratory approach, characterized by student-initiated learning (Berre et al., 2024; Jensen et al., 2018). Therefore, this paper additionally draws on insights from existing research that is more evaluative, in terms of educator-initiated learning activities. Bingen et al. (2019) found that first year nursing students often struggled to adapt to what they refer to as self-regulated learning. Most students preferred using videos from an external learning resource rather than the internally available online resources within the blended learning design, and especially appreciated saving time using online resources that covered all the knowledge they needed to pass the exam (Bingen et al., 2019).

Guy et al. (2018) explored optional bioscience e-learning resources among first year nursing students. Students reported interactive video clips engaging and helpful for their understanding of bioscience content. Moreover, the authors found that deep learning approach and subject scores were higher among the students who accessed the optional online resources, than among those who did not access these resources (Guy et al., 2018). Noteworthy, all groups in the study showed a decrease in deep-learning motive during the semester, which the authors relate to the complexity of the workload. Furthermore, the authors highlight a positive link between deep learning approach and access to optional online resources and imply a need to support first year students in developing what they refer to as self-regulation skills (Guy et al., 2018). An et al. (2022) conducted a Pilot Randomized Controlled Trial to compare the effects of what they refer to as self-regulated learning using augmented reality (AR) and the textbook among nursing students studying anatomy. The authors found that competency to self-regulate their learning improved more in the textbook group, implying that use of innovative educational resources was not superior to the textbook in enhancing self-regulated learning competency. However, the authors noted the short timespan (four weeks) of their study as a potential limitation in improving self-regulated learning competency.

Studies suggest that while digital resources in bioscience in nurse education may offer opportunities for self-directed learning (Egilsdottir et al., 2021; Evensen et al., 2020; Montayre & Sparks, 2018), students’ abilities to engage with these resources effectively vary (Evensen et al., 2020; Guy et al., 2018). Additionally, broader inquiries are needed to better understand what students experience as supportive in their use of digital learning resources (Harasim, 2017) and what kind of digital resources or tools can enhance students’ learning in bioscience outside formal lectures (Knutstad et al., 2021). These findings from previous research, combined with the pedagogical emphasis on lifelong learning within the context of digitalisation in higher education, highlight the need to explore how digital resources can support self-directed learning. Gaining such insights is beneficial for nurse education to better support students in developing self-directed learning skills in subjects such as bioscience as they are skills that form the basis for ongoing education and professional nursing knowledge. Hence, the objective of this study is to explore how digital learning resources can support self-directed learning among first year nursing students in bioscience.

### Theoretical Framework

**Figure 1.**
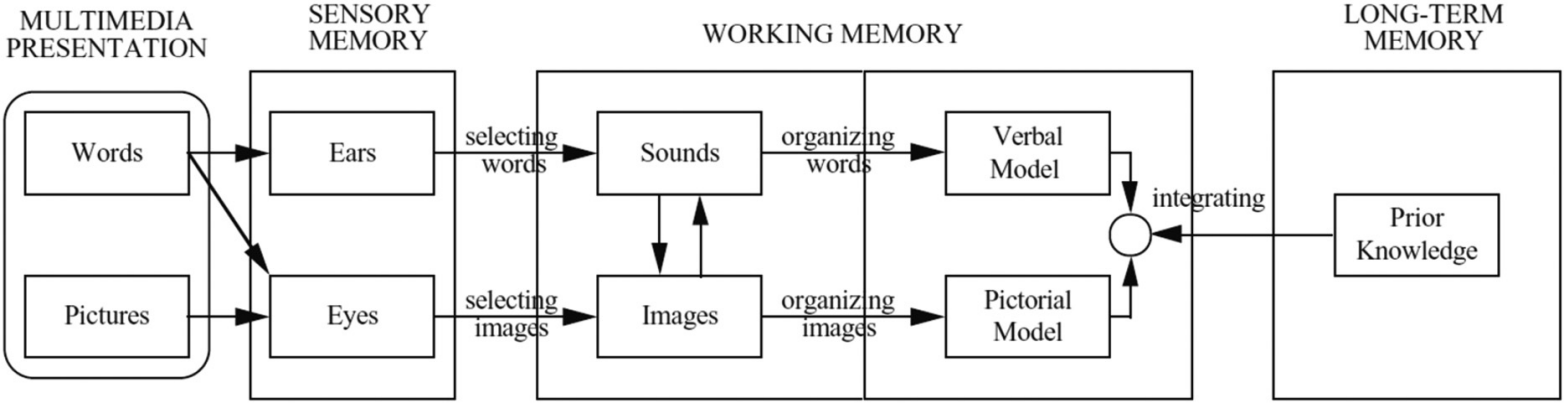
The Cognitive Theory of Multimedia Learning (Mayer, 2022, p. 62).

Drawing on previous research that highlights the value of pedagogically grounded integration of digital resources (Korseberg & Stalheim, 2024), the researchers strive to discuss the findings through the lens of Mayer's cognitive theory of multimedia learning (CTML) (Mayer, 2022). Inspired by Sweller's cognitive load theory, among others, CTML is based on how we process information, assuming that people learn more deeply from a combination of words and graphics, hence, referred to as multimedia (Mayer, 2022). Viewing multimedia as knowledge construction, deep learning is understood as the learner's ability to make sense of the presented information, and integrate mental representations from words and pictures into coherent understanding (Mayer & Fiorella, 2022). The CTML is based on the principles of *selecting, organizing and integrating information*, and is underpinned by the three assumptions ‘dual channels’, ‘limited capacity’ and ‘active processing’ (see Figure 1) (Mayer, 2022). ‘Dual channels’ assumes that humans process information through two separate channels, depending on the way information is represented: The visual channel processes information presented to the eyes, such as illustrations, videos and on-screen text, whereas the auditory channel processes information presented to the ears, such as sounds (Mayer, 2022). CTML assumes that each channel has ‘limited capacity’ to hold information at any one time in the working memory, requiring us to select pieces of information we deem important, and to what extent the selected information is to be connected with each other and existing knowledge into the long-term memory (Mayer, 2022). Such organizing methods are what Mayer (2022) refers to as metacognitive strategies. ‘Active processing’ is the active cognitive process in which humans build ‘coherent mental representations’ by selecting important information, organizing that information into meaningful structures and integrating information with other knowledge (Mayer, 2022). The feedback principle in multimedia learning is important for students to be able to make sense of information (Johnson & Marraffino, 2022). For feedback to be effective in a multimedia context, it should emphasize the learning process and development of metacognitive thinking for novice students, rather than focusing on the learning task alone (Johnson & Marraffino, 2022).

## Method

### Study Design

A qualitative, exploratory approach utilizing individual interviews was employed. The exploratory design aligns with the study's objective, which is to explore how digital resources can support self-directed learning among first year nursing students in bioscience. The researchers’ interest in this study lies in understanding the students’ personal perspectives and experiences, for which a qualitative method is a suitable approach (Braun & Clarke, 2022; Malterud, 2001).

### Research Question

How can digital resources support self-directed learning among first year nursing students in bioscience?

### Sample

Approximately 600 first year students at one Norwegian university were invited to participate in the study. A total of 16 students agreed to take part in the study and individual interviews were conducted with each student. The participants consisted of 4 males and 12 females, aged between 18 and 30 years (see Table 1).

**Table 1.** Overview Over Participants and Relevant Background, Grouped by Age.

Age	Participants	Previous bachelor's degree, relevant for nursing education	Previous work experience within health care
18–20	9		
21–25	6	1	1
26–30	1		1

Given the students’ range of backgrounds and previous experience, this study groups them by age and general background themes to ensure the protection of their identities.

The participants were recruited by the first author through oral invitation presented by visiting bioscience lectures and seminars. The first author presented the study and left written information and a list where the students could report their interest in participation. The written information concentrated on the study as well as the researchers’ academic affiliations and interests in the research topic. The bioscience teachers encouraged and reminded the students about the study before class ended, and the teachers collected the lists and handed them to the researcher. Seventy students reported their interest, and the researcher contacted each by e-mail individually, to ensure privacy protection. Sixteen students responded and gave their written consent to participate in individual interviews. The first author and the participants used e-mail to communicate information and organize time and place for interviews. Participants were later given the opportunity to read the transcription of their interviews. Three participants wished to do so and were sent their transcription via e-mail. None had any comments on their transcribed interviews. The e-mail correspondence was deleted after this.

### Inclusion Criteria

All first-year nursing students enrolled at one university in Norway.

### Data Collection

Individual interviews are used as a research method within health science to gain in-depth knowledge through systematic conversations (Brinkmann & Kvale, 2015). Such interviews seek nuances and detailed descriptions about the participant's experiences, thoughts, attitudes and beliefs (2015).

The data collection took place in the period September to December 2019, in parallel with the bioscience course. The interviews were performed using a semi-structured interview guide, informed by a pilot interview with two third year students. The interviews took place at the university. The interview first focused on themes such as the students’ background and motivation for choosing nurse education and their thoughts on bioscience as a part of nursing knowledge. The interview contained detailed questions about students’ study methods, preferences regarding digital resources, and experiences and habits regarding self-directed learning. A reference group consisting of two supervisors, two third year nursing students, two teachers responsible for the bioscience course and an academic librarian evaluated the interview guide before the data collection began.

The interviews were conducted by the first author and lasted between 35–57 min. First author made notes during each interview for later recall of ambience, context, participants’ tone of voice etc. Each interview was audio recorded and transcribed verbatim in Word by the first author. The transcribed files were exported into NVivo for coding and organization of the data.

### Data Analysis

The data analysis is conducted using Braun and Clarke's six phases of reflexive thematic analysis (2022). In the first phase ‘getting familiar with data’, first author listened to recorded interviews, made notes, and transcribed audio records in Word. The transcribed interviews were printed and re-read. In the second phase, ‘coding’, the first author coded the entire dataset and organised data in NVivo. All authors had full access to NVivo and contributed to reading of transcriptions. In the third phase, ‘generating initial themes’, the first author clustered codes to create candidate themes addressing the research question. The candidate themes were labelled ‘overwhelmed by bioscience content’, ‘guided learning with digital resources’, ‘learning towards exam’ and ‘acquiring in-depth knowledge challenging’. In phase four ‘developing and renewing themes’, the author group explored and addressed the candidate themes from phase three. The author group further discussed how candidate themes could be merged and split into new themes. The candidate themes addressing overwhelm and guided learning were merged into the first theme as presented in this study. The candidate themes addressing exam and in-depth knowledge were merged into the second theme as presented in this study. Renewed themes were contextualised with existing literature addressing the empirical data. In phases three to four, the Cognitive Theory of Multimedia Learning was introduced as a guiding theoretical framework after the researchers discovered its relevance to the findings (e.g., educational videos representing the combination of words and graphics and the interest in students’ sensemaking of the information through the digital resources). This theoretical perspective was utilized to contextualize the findings and informing further analysis. In phase five, ‘refining, defining and naming themes’, the researchers clarified content of themes, and named them to represent their patterns. The final themes and quotes were checked with transcripts to ensure they represent the correct interview context and coherence. During the last phase, ‘writing up’, the researchers checked metadata for variations in findings representation, shaped the analysis and wrote the article. Braun and Clarke (2022) emphasize that these phases are recursive, meaning the researchers move back and forth between all different phases, which is also the case for the conduction of the analysis in this study. Additionally, balancing between inductive (data-oriented) and deductive (theory-oriented) analytical approach as described here is common when doing RTA (Braun & Clarke, 2022; Byrne, 2022). This way, reflexive thematic analysis allowed the influence of CTML as a theoretical framework alongside the development of themes.

The reflexive thematic analysis resulted in two main themes and six subthemes, as shown in Table 2.

**Table 2.** Themes and Subthemes Developed from Reflexive Thematic Analysis.

Theme	Subtheme
1. Digital resources provide an overview over bioscience	Feeling of control Guided learning Challenges with learning bioscience
2. Motivational issues – deep learning or passing the exam	Deep learning main goal Lowering ambitions Passing exam achievable

### Ethical Considerations

Ethical approval for this research was obtained through Sikt - Norwegian Agency for Shared Services in Education and Research. All participants were given oral and written information and provided written informed consent to participate and publish prior to participating. They could withdraw from the study at any time without consequences. All participants were given numbers in the researchers’ notes to protect their identity. The recorded interviews were only recognizable through metadata, which were kept locked in the researcher's office. As soon as the interviews had been recorded, the information was transferred to an encrypted memory pen via an offline computer, and the recorder was immediately reformatted. Information about participants was stored locked away in the first author's office as handwritten notes and an encrypted memory pen.

The researcher conducting the interviews communicated with the participants via their student e-mail address. The students were all contacted individually to make appointments regarding time and date of the interview. Written consent forms were collected by hand at the beginning of each interview and were kept locked in the researcher's office.

## Results

This section highlights findings from interviews with first year nursing students about their use of digital learning resources in self-directed learning in bioscience. The findings are presented as the two main themes developed from the data analysis: ‘digital resources provide an overview over bioscience’, and ‘motivational issues – deep learning or passing the exam’. These qualitative findings, despite being presented as separate themes, will naturally overlap somewhat.

### Digital Resources Provide an Overview Over Bioscience

Nursing students use of digital learning resources is often described in relation to the challenges they encounter in the bioscience subject. It was recurring that first-year nursing students felt overwhelmed by the subject's requirements, e.g., amount of content and difficult scientific terms. A frequently addressed challenge among the students in this study was to distinguish important and not so important knowledge from their learning resources: ‘*I can very well begin with highlighting in the book, but it's so difficult to know what is important and what's not important’* (13).

The students in this study used digital learning resources in the form of online videos and illustrations in combination with audio presentations, online interactive problem-solving and quizzes. Many students found help in educational videos to prioritize content. There are both institutional and non-institutional online platforms providing educational videos in the bioscience subject. Most students found the non-institutional digital resources helpful in providing an overview of what they were supposed to learn: ‘*The* (non-institutional) *teacher explains everything, really. I would have never managed to figure out all the things they say by myself. So, when they say, “this is important to know”, and “this is not important to know”, I think “wow, that's so good to hear!”, because I would have never figured that out myself’* (1).

The non-institutional online platform offers bioscience content specifically targeted to health profession students. This resource allows students to solve problems related to each topic, ask questions, and receive feedback on their efforts. Students described this resource as straightforward and appreciated its guidance: ‘*…You know exactly what you’re supposed to learn and what level you’re on’* (15). Students commonly appreciated such ‘recipe’ to study bioscience, and underlined how bioscience went from chaos to achievable when following the ‘recipe’ this resource provided: ‘*I believe you are more in control using this resource, like, I know how to work with it. In class, it's like “now you should be done with these chapters”, and if I were to follow that, I would try out so many different ways to work with it, and I would never have managed to get through it all’* (9).

In extension to the feeling of control with digital resources, receiving feedback was experienced as engaging and motivating for further studies: ‘*It's the feedback you get, that you need to watch this many videos to complete a topic, like, personal follow-up. It's like there's someone there pushing you a little, and that gets to me more than when university teachers state how far we should have come by now. That's just stressful’ (7).*

Another advantage the students mentioned was the language used in the digital resources, which seems to have been somewhat easier to understand than other resources: ‘*…they give us both the “kid's version” and the “grown-up version”… I also need the “grown-up version” so I’m absolutely certain what is correct, … but to understand it, it's very good using the “kid's version”’* (14).

The students also frequently searched Google or medical encyclopaedia for information, aiming for a short and understandable description. They often searched terminology or sequences of physiological processes that they did not understand using other learning resources: ‘*I type in the word I’m looking for + meaning. … Then I read for example the medical encyclopaedia. They provide brief and good explanations’* (12).

One of the campuses video-recorded bioscience lectures, which were appreciated by the students who were not satisfied with the non-institutional resource; ‘*Well, many of my peers use that* (non-institutional) *resource, and I sat with them once and listened, but I think it was very simplified. So, for me, it's better to listen to the recording from class, and just fast forward past what I don’t need. Since I was in class, I know what I did and didn’t understand’* (10).

Regardless of which digital resource was used, the online platforms offered a flexible, self-paced approach to learning: ‘*What's nice with that is that you can pause, fast forward and rewind. That's not possible in the auditorium, where it all often goes too fast’* (11).

For the nursing students in this study, learning challenges were related to feeling overwhelmed by the requirements in the bioscience subject and difficulty knowing how and what content to select and how to structure their learning process. The main advantages of using digital resources included knowing what to learn, getting a better overview over bioscience content and receiving personalized feedback.

### Motivational Issues–Deep Learning or Passing the Exam

The students have given rich reflections on the need for learning bioscience thoroughly to understand how the human body works to identify symptoms and illness and to provide safe and compassionate care to patients and their families. Many express awareness of their responsibility in practicing patient care, and that in-depth knowledge in bioscience will help them assemble comprehensive knowledge in both upcoming subjects and in clinical practice. Yet, it seems to be conflicting interests between the motivation for deep learning and preparing for the final exam. In this regard, digital resources seemed to be mainly used strategically as a tool to manage the requirements of exam performance: ‘*I think the most important thing is to learn… But everyone gets so focused on passing and doing well on the final exam, because that's what counts the most. But it is important also to understand it all properly’ (1).* A common response to the demands of the bioscience subject was to lower one's ambitions for the upcoming exam. High-achieving students described: ‘*The way it is now, it's so much and extensive, and we have two others, quite large, subjects at the same time. So now my focus is just really to pass. I mean, I want good grades, but I’m not aiming for that A. I want to, but my focus is to pass’ (4).*

The students’ appreciated digital resources that highlighted what was needed to achieve good results on the final exam: ‘*The teacher illustrates that “this usually comes up on the exam”, so now I take screenshots of that. For example, the* (non-institutional) *teacher will say “if you know this, you will pass. If you also know this, you will get a C. If you want an A, you need to know this”. I have taken screenshots of that, because it is so well formulated, and it really gives me a lot’* (12).

When the students were asked to answer in retrospect, how they would approach learning bioscience if the semester restarted ‘tomorrow’, nearly all the students stated that they would structure and begin studying even earlier in the semester. For many, the first weeks of the semester were largely affected by not knowing how to study or where to find information: ‘*I wish I knew that the digital resource helped me the way it actually does! If I had known, I might have worked more effectively from the beginning instead of sitting there like “ah, using this book just doesn’t work, what should I do?”’ (15)*.

Overall, students emphasize the value of developing a deep understanding of bioscience. However, this goal seems to conflict with the focus on performing well on the final exam. Among first year-students in bioscience, self-directed learning and the use of digital resources appear to be driven by the demands of subject assessment rather than by their motivation for deep learning.

## Discussion

In this study, two themes are reported that provided insight into how digital resources can support self-directed learning in bioscience among first year nursing students. In this section, the findings are discussed in light of previous research and Mayer's Cognitive Theory of Multimedia Learning (Mayer, 2022). While the current study is conducted in Norwayand a self-directed learning context, the findings may be of relevance to other educational settings and countries as well, as digital resources are increasingly used in higher education teaching and learning across different educational settings (Korseberg & Stalheim, 2024). Considering the importance for nurses to understand bioscience in order to ensure safe patient care, our findings may extend to various educational settings. The findings highlight how digital resources can be used to support students’ self-directed learning in this important area.

The students in this study felt overwhelmed by the demands of the bioscience subject, consistent with previous research which has found that nursing students often experience feelings of being overwhelmed when entering nurse education and facing the learning requirements (Bingen et al., 2019; Torbjørnsen et al., 2021). This current study adds to this body of knowledge with findings that digital resources offering a visual overview of bioscience content and subject requirements along with personalized feedback seem to give students a sense of control in their self-directed learning and thereby may ease their feelings of overwhelm. These findings can be understood as supportive of self-directed learning, as they may reflect students’ developing ability to navigate and manage their own learning processes.

In this way, the study contributes to the field by identifying specific aspects that first year students’ experience as supportive in their use of digital resources for self-directed learning in bioscience. Liu et al. (2023) found that students perceived institutional technologies as being overloaded with information and turned to non-institutional resources to control their own learning which supports the findings of this current study. This can be one way that digital resources can create value beyond merely using it to “make educational practice easier, scalable or distributed” within health professions education as pointed out by Grainger et al. (2023, p. 7). Regarding the emphasis on lifelong learning and digitalisation in higher education (Lindqvist et al., 2024), students need to manage their own learning processes outside the formal lecture settings. Additionally, they need competence in navigating, choosing and monitor their own learning using digital resources. This is supported by Cramarenco et al. (2023) who recommend future directions in education in light of digitalisation, highlighting a growing need for pedagogical approaches that go beyond content delivery, and which emphasizes students’ self-directed learning.

The integration of Mayer's Cognitive Theory of Multimedia Learning (CTML) in this study offers a theoretical lens to understand how digital resources can support self-directed learning; The educational videos in the online platforms combined visual and auditory material, understood as multimedia material, which students found supportive for gaining an overview of bioscience content, concentrate on learning requirements, and help them decide what to learn, and where to find learning materials, in accordance with Duval et al. (2017). In light of CTML (Mayer, 2022), this is an interesting finding, as reducing cognitive load is central to promote meaningful learning. However, multimedia materials are most helpful for deep learning when they are clearly structured and guides students in organizing and integrating information. If the digital resources lack guidance or is presented as isolated facts, the students may feel overwhelmed (Mayer, 2022). The students seem to experience motivational issues and tend to use digital resources primarily for assessment purposes, such as achieving a specific grade on final exam. However, for multimedia learning to be helpful for deep learning, as Mayer (2022) describes, the learning material must be designed in a way that aligns with how human process information, not only by combining words and graphics to provide supportive features such as visual overviews, but also to guide the students in active processing of the information. In this way, CTML may guide the design of digital resources to support deep learning among higher education students.

Interestingly, Johnston et al. (2018) highlight that the use of video in bioscience learning, founded within a curriculum structure, can create a self-paced, student-centred, motivating learning process. Egilsdottir et al. (2021) found that institutional digital multimedia learning resources added value to development of knowledge and self-efficacy in learning and integrating bioscience knowledge. These are interesting nuance to the findings of this current study, where the students primarily seemed to experience support in digital resources by providing an overview of bioscience content and guidance in how and what to study. In this study there also seemed to be conflicting focuses on exam and deep learning, in line with the findings of Bingen et al. (2019). These nuances may be partially explained by the different study contexts; this study being exploratory, whereas Egilsdottir et al. (2021) and Johnston et al. (2018) focused on digital resources grounded within an institutional setting and evaluation of these. The nuances might also reflect the broader insights from educational research, indicating that the potential of digital resources to support learning depends more on how they are pedagogically integrated into the subject, rather than on the technology itself (Cramarenco et al., 2023; Grainger et al., 2023; Korseberg & Stalheim, 2024). In extension, researchers have argued that metacognitive learning skills among nursing students in bioscience are more important than the digital resources themselves, as effective use of such resources requires students to understand how to use them to support their learning (Berre et al., 2024). This is similar to the discussion of Lindqvist et al. (2024) that highlights the need for learning to learn for students to benefit from the possibilities within lifelong learning. It is possible that multimedia materials through digital resources are delivered in different ways across institutional and non-institutional settings, with varying considerations of pedagogically approaches and metacognitive learning, with respect to this aspect requiring further research.

In the current study the authors interpret that digital resources may support self-directed learning by helping students identify and select important bioscience content. For students to be able to make sense of learning material and achieve deep learning through using digital resources in self-directed learning, metacognitive strategies seem to be a prerequisite, accompanied by the principles of selecting, organizing, and integrating information (Mayer, 2022). Duval et al. (2017) state that especially young learners need assistance in developing the metacognitive skills needed to succeed with learning with digital resources on their own. The consensus that feedback in education is effective, as it provides students with information about their learning progress is challenged within the context of multimedia learning (Johnson & Marraffino, 2022). In light of CTML, the effect of feedback depends on the learning goal, that is, to remember or to transfer knowledge. In multimedia learning, feedback should also encourage metacognitive thinking, which is necessary to integrate knowledge and understanding. Feedback has been shown to have limited effect in learning when provided without these considerations (Johnson & Marraffino, 2022). It might be that the feedback, in which the students experience personalized, from non-institutional digital resources enhances exam-focus more than promoting metacognitive learning skills in bioscience when presented as what students need to know to achieve certain grades. On the other hand, it is worth acknowledging the reasonable assumption that nursing students experience such feedback as valuable in their self-directed learning in bioscience.

Another aspect of how students’ may experience support in digital resources for self-directed learning may be that such resources provide the accustomed flexibility of technology in everyday life and the students may expect to process their education with the same technological habits. This way, students may prefer digital resources offering a format they already know when searching for understanding. Molin et al. (2021) found that first year nursing students studying bioscience tend to use the resource they themselves find most attractive. However, students’ satisfaction with digital resources in bioscience seems to have no correlation with achievement (Jensen et al., 2018). This gap between satisfaction and achievement might also reflect the interpretation of issues between deep learning and passing final exam, similar to Bingen et al. (2019).

### Strengths and Limitations of the Work

This qualitative study shares insights from interviews conducted prior to the COVID 19-pandemic. The authors believe that these insights, especially in light of CTML, are relevant following the increased use of digital resources in education after the pandemic. The authors posit that the participants’ descriptions of engaging with digital resources for their self-directed learning are genuine and driven by their individual needs, and are not influenced by unforeseen circumstances, such as the pandemic.

The authors have placed emphasis on CTML as a theoretical lens to assemble a broader understanding of how nursing students experience support using digital resources for self-directed learning. However, this interpretation and discussion should be viewed as an initial exploration. Despite this, the findings may point to some practical implications: digital learning resources providing a structured overview of subject content and providing guidance on what content to select may be experienced as supportive by first year nursing students in their self-directed learning. However, based on previous research and the theoretical framework in this current study, the authors argue for the importance of considering metacognitive aspects into the design of such resources. In this regard, the pedagogical principles outlined in the CTML may be valuable.

The authors have worked in a team, interpreting, and analysing data. Elements of the study, such as the interview guides, have been discussed with the reference group as described, and the study has been discussed throughout its progress with the research group in education. The authors consider these factors as strengths of the study.

### Implications for Practice

CTML may offer valuable insight into how digital resources can be designed to enhance understanding in bioscience, which ultimately may support the development of lifelong learning among nursing students. For bioscience educators, this suggests practical implications such as applying the principles of selecting, organizing and integrating information to reduce cognitive load and enhance students’ ability to process and connect complex concepts. Educators can also use these principles to guide first-year students in navigating bioscience content and managing self-directed learning processes more effectively to support students’ efforts in understanding bioscience. Based on the findings in this study, the authors recommend further research that expands the understanding of how digital resources can best support self-directed learning in bioscience. Particularly, more studies are needed to explore the pedagogical use of digital resources in ways that align with theoretical perspectives such as CTML or other suitable frameworks. This alignment would help bridge the gap between the use of digital resources and pedagogical principles, ensuring that the digital resources are not only accessible but also pedagogically effective in supporting students’ learning.

## Conclusion

This study contributes to a better understanding of how digital resources can support self-directed learning among first year nursing students in bioscience, especially through guidance in studying bioscience. Such guidance helps students gain a better overview over complex content, select relevant information and gives the students a sense of control over their learning process. While existing literature remains cautious about digital resources leading to learning itself, there is broad agreement that its impact depends on how it is pedagogically integrated into the course design. By applying the Cognitive Theory of Multimedia Learning, this study highlights the importance of critically evaluating how digital resources can be structured to promote support for students’ self-directed learning. The support that students’ experienced by using digital resources is worth taking into consideration for further research and development of multimedia teaching- and learning design.
